# Association of MicroRNA-155rs767649 Polymorphism with Susceptibility to Preeclampsia

**DOI:** 10.22088/IJMCM.BUMS.8.4.247

**Published:** 2019

**Authors:** Shymaa E. Ayoub, Olfat G. Shaker, Mostafa Y. Abdelwahed, Naglaa A. Ahmed, Hazem G. Abdelhameed, Almandouh H. Bosilah, Sheren R. Mohammed

**Affiliations:** 1 *Department of Biochemistry and Molecular Biology, Fayoum University, Al Fayoum, Egypt. *; 2 *Department of Medical Biochemistry and Molecular Biology, Faculty of Medicine, Cairo University, Giza, Egypt.*; 3 *Department of Physiology, Faculty of Medicine, Fayoum University, Al Fayoum, Egypt.*; 4 *Department of Physiology, Faculty of Medicine, Zagazig University, El Zagazig, Egypt.*; 5 *Department of Gynecology and Obstetrics, Faculty of Medicine, Fayoum University, Al Fayoum, Egypt.*

**Keywords:** MicroRNA, mir-155, single nucleotidepolymorphism, preeclampsia

## Abstract

Preeclampsia (PE) is a multifactorial disorder. Several studies showed that micro RNAs may play a critical role in PE pathogenesis. We aimed to investigate for the first time the association of mir-155rs767649 polymorphism with PE. Eighty patients with preeclampsia and 80 normal subjects were enrolled in the study. Serum expression levels of mature mir-155were evaluated using real-time PCR, and mir-155 rs767649 (T/A) polymorphism was genotyped using TaqMan SNP genotyping. There was a significant difference between the expression level of mir-155 in cases (5.86 ± 3.11) in comparison with controls (0.58 ± 0.30) (P<0.0001)**. **Also,the minor allele of rs767649 was significantly associated with increased risk of PE [Recessive model: adjusted Odds ratio (OR) = 5.240, 95% confidence interval (CI) = (1.999-13.733),P= 0.001]. There was a significant difference between different genotypes according to expression levels of mir-155 in PE (P<0.0001) with high expression levels in TA genotype (7.10 ± 3.11 ). Mir-155 may play a critical role in PE pathogenesis. The obtained data suggest that a minor allele of rs767649 might be a predisposing factor for PE.

Preeclampsia (PE) is a pregnancy-related disorder characterized by the development of hypertension and proteinuria after 20 weeks of gestation (1). It represents about 2- 5% of pregnancies worldwide, and causes 10% to 15% of maternal deaths (2).The placenta plays an important role in the initiation and progression of the disease (3). The condition starts by the impaired extravillous trophoblasts proliferation and invasion accompanied by poor spiral vascular remodeling leading to decreased blood flow into the intervillous space causing placental underperfusion (4). Placental pro-inflammatory and antiangiogenic factors released in the maternal circulation, cause maternal systemic endothelial cells dysfunction and systemic inflammation (5).Reported risk factors include maternal age, nulliparity, diabetes, and hypertension (6). The condition could be complicated by elevated liver enzymes, hemolysis and low platelet count syndrome or eclampsia with visual disturbances and seizures (7).

MicroRNAs (miRNAs) represent a subgroup of non- protein-coding RNAs that are short (about 22 nucleotides) and highly conserved. They are key regulators of gene expression by destabilizing mRNAs or down-regulating their target genes (8). Evidence proved that mir-155 was up- regulated in the placenta from numerous pregnant women suffering from PE. Mir-155 can be significantly up-regulated by tumor necrosis factor and lipopoly-saccharide, and it can regulate nuclear factor (NF)-kB(9).

Single nucleotide polymorphisms (SNPs) are DNA sequence variations that can interfere with posttranscriptional activities such as protein binding, polyadenylation, and miRNA binding. So, they can affect gene regulation (10). The rs767649 polymorphism in the promoter of mir-155 was reported in many diseases such as cervical cancer (11), hepatocellular carcinoma, (12) and lung cancer (13).

We aimed to detect the expression level of mir-155 and the association of mir-155 rs767649 polymorphism with PE.

## Materials and methods

**Subjects**

Our study included 160 subjects divided into 80 pregnant women with recently diagnosed preeclampsia before taking any treatment, and 80 women with normal pregnancies that were selected as controls, and were sequentially collected from outpatient clinics and inpatient Department of Obstetrics and Gynecology, Fayoum University Hospital, Egypt.The study was revised and approved by the Faculty of Medicine, Fayoum University Ethical Committee, and written in-formed consent was obtained from all pregnant women before sample collection. PE was diagnosed according to the standard criteria: systolic blood pressure> 140 mmHg and/ or diastolic blood pressure> 90 mmHg on two occasions at least, accompanied with a urinary protein level>0.3 g in a 24 h urine collection. All subjects were unrelated and of the same race. Pregnant women with any other complications including maternal history of renal disease and/ or hypertension, diabetes, smoking, chromosomal abnormalities, alcoholism, and fetal congenital abnormalities were excluded from our study.

**Samples collection**

Six ml blood was withdrawn and collected in 3 tubes.One of them being a plain tube that was allowed to clot for 15 min, and centrifuged at 4000g for 10 min. Serum samples were separated and stored at -80^o^C until use. These sera were used forbiochemical analyses, and mir-155 expression evaluation. One tube contained sodium citrate for prothrombin time (PT) measurement. The third tube contained EDTA and was stored at -80^o^C until DNAextraction and genotyping of the studied SNP(rs767649) using real-time polymerase chain reaction (PCR).

The sample size was calculated according to Epi Info2000, a special formula used based on the prevalence of disease at a confidence interval (CI) of 95% and a precision of 2%. The sample size was increased by 10% to overcome problems related to missing data.

**RNA extraction and reverse transcription rea-ction**

RNAs were extracted from all samples using (Qiagen, Germany) RNA extraction kit, and reverse transcribed into cDNAs using (Qiagen, Germany) RT-PCR kit according to manufacturer’s instru-ctions.

**Real-time PCR**

The serum expression level of mir -155 was evaluated using the miScript SYBR Green PCR Kit (Qiagen, Germany). Primers of mir-155 and internal control were obtained from Qiagen, Ger-many (MS00033712). MiRNA *SNORD68* was used as internal control. Real-time PCR was performed using Rotor-gene Q System (Qiagen, Germany). The relative expression of RNA was calculated by the 2^-ΔΔCt^ method for relative quantification (14).

**Genotyping**

DNA was extracted from whole EDTA blood samples using the QIAamp DNA MiniKit (Qiagen, Germany). Mir-155 rs767649 (T/A) polymorphism was genotyped using TaqMan SNP Genotyping assay. DNA amplification was performed using a Rotor gene Q System (Qiagen, Germany).

**Statistical analysis**

Statistical analyses were performed with SPSS V 20. Demographic differences between groups were examined by Mann- Whitney U and Chisquared (χ2) test. The correlation of study parameters was examined by Spearman correlation. The frequencies of the alleles and genotypes were analyzed by the (χ2) test. The odds ratio (OR) and 95 % confidence intervals (CI) were also estimated by using logistic regression analyses to evaluate the associations between genotypes and PE with adjustment for age and body mass index (BMI). Data were presented as the median. A comparison between genotypes was done by Kruskal-Wallis and Chi-squared (χ2) test. The value of P < 0.05 was considered as statistically significant.

## Results

**Demography and laboratory characteristics of the study groups**

Table 1 shows that there was a significant difference between subjects and controls regarding parity (P= 0.014), systolic blood pressure (SBP), diastolic blood pressure (DBP), mean arterial pressure (MAP), aspartate transaminase (AST), alanine transaminase (ALT), C-reactive protein (CRP), urine albumin, and creatinine with P<0.0001 for each. Direct bilirubin (P = 0.001), alkaline phosphatase (ALP) (P= 0.025), and serum uric acid (P= 0.005) showed higher levels in PE patients. fasting blood sugar (FBS) (P= 0.001), 2 hpostprandial(PP) (P= 0.012), and prothrombin concentration (PC) (P= 0.009) showed higher levels among controls. Table 1 shows also that 40% of cases had a mild degree of the disease while 60% had a severe degree.

**MiR-155 expression levels in preeclampsia patients**

There was a significant difference between the cases and controls regarding the expression level of mir-155 with up-regulation in PE patients (5.86 ± 3.11) in comparison with controls (0.58 ± 0.30) (P<0.0001) (Table 1).

**Correlation of the expression levels of mir-155 with study parameters among cases**

Spearman correlation among study parameters in cases showed that there was a positive correlation between the expression level of mir-155and age (r =0.002), gravity (r=0.006), parity (r=0.045), abortion (r=0.003), fetal birth weight (r=0.036) while it showed negative correlation with FBS (r=-0.036) (Table 2).

**Genotypes and alleles frequencies of rs767649**

Logistic regression analysis revealed that the minor allele of rs767649 was significantly associated with increased risk of PE (recessive model: adjusted OR = 5.240, 95% CI = 1.999-13.733, P = 0.001) after adjusting for age, BMI. A allele was significantly associated with PE risk, compared with the T allele (OR=1.751, 95% CI=1.112-2.757, P = 0.016) (Table 3).

**Basic and laboratory characteristics for different rs767649 genotypes in preeclampsia cases**

Table 4 shows basic and laboratory characteristics for different genotypes of rs 767649. No difference between the three genotypes was observed regar-ding gravidity, parity, abortion, AST, albumin, total bilirubin, indirect bilirubin, urineal-bumin, urineglucose, urea, serum uric acid, FBS,2 h PP, PC, and degrees of PE. Meanwhile, there were significant differences between different genotypes according age, BMI, SBP, MAP, direct bilirubin, PT, DBP, ALP, ALT, CRP, serumc-reatinine, partial thromboplastin time (PTT), and international normalized ratio (INR).

**Table 1:** Distribution of study groups according to their basic and laboratory characteristics

**P-value** ^#^	** Patients (N=80)**		**Controls (N=80)**	**Variables**
	**Median (range)**			
0.460	30.5 (21-41)	32 (19-42)		**Age (years)**
0.443	31.5 (25-38)	30.5 (24-41)		**BMI**
0.421	1 (0-8)	2 (0-9)		**Gravidity**
**0.014** ^*^	1 (0-6)	2 (0-5)		**Parity**
0.361	0 (0-3)	0 (0-7)		**Abortion**
**<0.0001** ^*^	110 (100-130)	160 (140-190)		**SBP (mmHg)**
**<0.0001** ^*^	75 (60-85)	110 (90-130)		**DBP (mmHg)**
**<0.0001** ^*^	93.75 (82.5-102.5)	132.5 (115-155)		**MAP(mmHg)**
**<0.0001** ^*^	14 (8-35)	25 (8-234)		**AST(IU/L)**
**<0.0001** ^*^	13.5 (7-39)	19.5 (9-269)		**ALT(IU/L)**
0.378	3.1 (2.8-3.8)	3.1 (2.3-3.7)		**Albumin(g/dL)**
0.364	0.4 (0.2-1.2)	0.5 (0.1-0.9)		**Total bilirubin (mg/dL)**
**0.001** ^*^	0.1 (0.01-0.4)	0.05 (0.01-0.4)		**Direct bilirubin(mg/dL)**
0.827	0.3 (0.1-0.9)	0.4 (0.05-0.8)		**Indirect bilirubin(mg/dL)**
**0.025** ^*^	74.5 (69-77)	75 (69-99)		**ALP(IU/L)**
**<0.0001** ^*^	8.5 (2-63)	30 (3-140)		**CRP (mg/L)**
**0.0001** ^*^	0 (0.0)	2 (1-4)		**Albumin in urine(g/dL)**
0.077	0 (0-1)	0 (0-1)		**Glucose in urine (mmol/L)**
0.547	25.5 (10-45)	23 (11-44)		**Urea (mg/dL)**
**<0.0001** ^*^	0.6 (0.3-1.1)	0.7 (0.5-1.5)		**Serumcreatinine(mg/dL)**
**0.005** ^*^	3.6 (3-4.8)	03.8 (3-7)		**Serumuric acid (mg/dL)**
**0.001** ^*^	86.5 (60-110)	77 (60-100)		**FBS (mg/dL)**
**0.012** ^*^	115 (90-158)	118.5 (95-145)		**2 h PP(mg/dL)**
0.132	13 (10-15)	13 (11-14)		**PT (s)**
**0.009** ^*^	113.5 (70-140)	90 (70-150)		**PC (mg/L)**
0.379	34.5 (26-44)	34 (24-45)		**PTT (s)**
0.322	1 (0.8-1.1)	0.9 (0.8-1)		**INR**
<0.0001*	0.58 (0.11-1.14)	5.02 (0.09-12.15)		**Relative expression level ** **of miR-155**
		Mild 32 /80 (40%)		**Severity of the disease**
		Severe 48/80 (60.0%)		

BMI:body mass index; SBP: systolic blood pressure; DBP: diastolic blood pressure; MAP: mean arterial pressure; AST: aspartate transaminase; ALT: alanine transaminase; ALP: alkaline phosphatase ;CRP: C-reactive protein; FBS: fasting blood sugar; 2 h PP:2h postprandial; PT: prothrombin time; PC: prothrombin concentration; PTT: partial thromboplastin time; INR: international normalized ratio.#: statistical analyzes were performed by the Mann-Whitney U and- Chi-squared (χ2) test.*: statistically significant

**Table 2:** Correlation of relative expression level of miR-155with study parameters among preeclampsia cases

**Mir-155relative expression level**		**Variables**
**P-value**	**r**	
**0.002** ^*^	**0.344**	**Age**
0.205	0.143	**BMI**
**0.006** ^*^	**0.306**	**Gravity**
**0.045** ^*^	**0.224**	**Parity**
**0.003** ^*^	**0.330**	**Abortion**
0.341	-0.108	**SBP**
0.571	0.064	**DBP**
0.775	-0.032	**MAP**
0.297	0.118	**AST**
0.979	0.003	**ALT**
0.156	-0.160	**Albumin**
0.852	0.021	**Total bilirubin**
0.725	-0.040	**Direct bilirubin**
0.366	0.102	**Indirect bilirubin**
0.365	-0.103	**ALP**
0.163	0.158	**CRP**
0.297	0.118	**Albumin in Urine**
0.053	-0.217	**Glucose in urine**
0.942	-0.008	**Urea**
0.443	0.087	**Serum creatinine**
0.123	-0.174	**Uric acid**
**0.002** ^*^	**-0.346**	**FBS**
0.868	0.019	**2h PP**
0.065	-0.208	**PT**
0.056	-0.215	**PC**
0.329	-0.111	**PTT**
0.177	0.152	**INR**
0.476	0.081	**Gestation week**
**0.036** ^*^	**0.235**	**Fetal birth weight**

BMI:body mass index; SBP: systolic blood pressure; DBP: diastolic blood pressure; MAP: mean arterial pressure; AST: aspartate transaminase; ALT: alanine transaminase; ALP: alkaline phosphatase ;CRP: C-reactive protein; FBS: fasting blood sugar; 2 h PP: 2h postprandial; PT: prothrombin time; PC: prothrombin concentration; PTT: partial thromboplastin time; INR: international normalized ratio.Statistical analyzes were performed by Spearman correlation test.*: statistically significant.

**Table 3:** Genotypes and alleles frequencies

**Adjusted** **OR** ^#^ ** (95%CI)** **P-value**	**Unadjusted** **OR (95%CI)** **P-value**	**Controls** **(N=80)**	**Patients** **(N=80)**	**Variables**
		**N (%)**		
**Genotype**				
1	1	12 (15.0)	8 (10.0)	TT
1.344 (0.497-3.633) 0.560	1.200 (0.454-3.171) 0.713	60 (75.0)	48 (60.0)	TA
6.811 (1.835-25.278) 0.004^*^	4.500 (1.355-14.944) 0.014^*^	8 (10.0)	24 (32.9)	AA
**Dominant model**				
1	1	31 (39.7)	9 (11.4)	TT
1.711 (0.648-4.518) 0.278	1.588 (0.612-4.123) 0.342	47 (60.3)	70 (88.6)	TA/AA
**Recessivemodel**				
1	1	74 (94.9)	53 (67.1)	TT/TA
5.240 (1.999-13.733) 0.001^*^	3.857 (1.611-2350.002^*^)	4 (5.1)	26 (32.9)	AA
**Allele**				
1	1	105 (67.3)	62 (39.2)	T
1.751 (1.112-2.757) 0.016^*^	1.658 (1.064-2.582) 0.025^*^	51 (32.7)	96 (60.8)	A

Logistic regression was applied with adjustment for age and BMI. *: Statistically significant

**Pregnancy and delivery characteristics for different rs767649 genotypes in preeclampsia cases**

Comparison of pregnancy and delivery characteristics for different genotypes in cases showed that there were significant differences between different genotypes according to gestational age (P <0.0001), fetal birth weight (P = 0.013), intra uterine growth retardation (P= 0.003)

(Table 5).

**Comparison of genotypes for expression levels of mir-155 in preeclampsia cases**

Table 6 showed that there were significant differences between the different genotypes according to the expression level of miR-155 in PE (P<0.0001) with high level in TA genotype (minmax) 7.47 (0.09-12.15) with p-value between different genotypes as follow (TT-TA: P= 0.007, TT-AA: P= 1.000, and TA-AA: P<0.0001).

**Prognostic performance of the best cut off values of serum mir-155 in preeclampsia group**

**Fig. 1:**
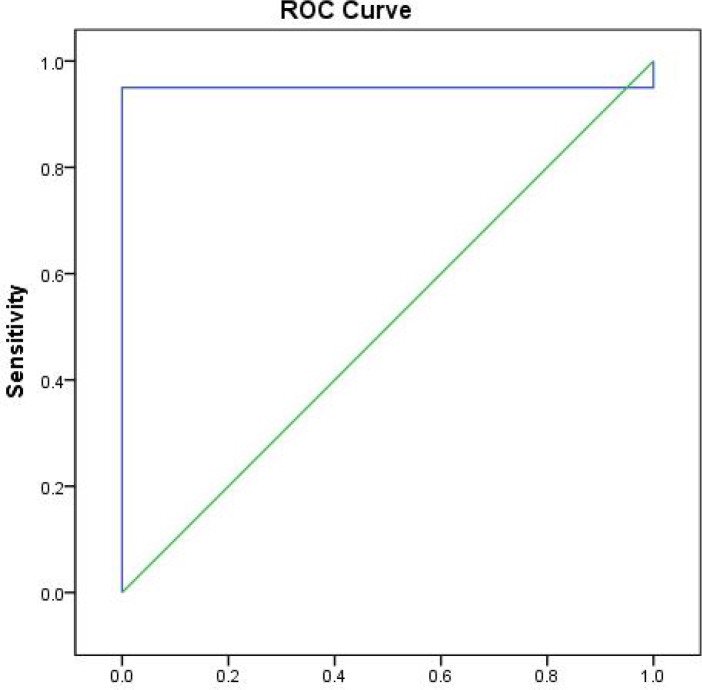
Receiver operating characteristic (ROC) curve of sensitivity and specificity of mir-155 between cases and controls.AUC: area under the curve

Figure 1 illustrates the ROC curve of mir-155 in PE group, showing the diagnostic value of this marker as a predictor in differentiating between cases of PE and controls as follows: area under the curve (AUC)= 0.950, P <0.0001, cutoff point 1.57, sensitivity 95.0%, and specificity 100.0%.

**Table 4:** Basic and laboratory characteristics in different genotypes in preeclampsia cases

**Variables**	**TT (N=8)**	**TA (N=48)**	**AA (N=24)**	**P-value** ^#^
	**Median (range)**			
**Age (years)**	25.5 (25-26)	33.5 (19-42)	31.5 (22-39)	**0.006** ^*^
**BMI**	29 (24-34)	28.5 (24-39)	38.0 (29-41)	**<0.0001** ^*^
**Gravidity**	1 (0-2)	2.5 (0-9)	2.0 (1-4)	0.075
**Parity**	1 (0-2)	2 (0-5)	1.5 (1-4)	**0.137**
**Abortion**	0 (0-0)	0 (0-7)	0 (0-1)	0.239
**SBP (mmHg)**	160 (150-170)	152.5 (140-180)	180.0 (140-190)	**<0.0001** ^*^
**DBP (mmHg)**	102.5 (95-110)	105 (90-130)	110 (100-130)	**0.020** ^*^
**MAP(mmHg)**	131.25 (122.5-140)	128.75 (115-155)	150.00 (120-155)	**<0.0001** ^*^
**AST (IU/L)**	35 (10-60)	22.5 (8-168)	27.0 (13-234)	0.241
**ALT (IU/L)**	31 (18-44)	18.5 (9-269)	23.0 (14-162)	**0.009** ^*^
**Albumin (g/dL)**	2.85 (2.6-3.1)	3.15 (2.3-3.7)	3.10 (2.6-3.6)	0.087
**Total bilirubin (mg/dL)**	0.46 (0.2-0.7)	0.55 (0.18-0.90)	0.4 (0.10-0.70)	0.179
**Direct bilirubin(mg/dL)**	0.02 (0.01-0.02)	0.06 (0.03-0.4)	0.05 (0.04-0.1)	**<0.0001** ^*^
**Indirect bilirubin(mg/dL)**	0.4 (0.19-0.69)	0.43 (0.1-0.84)	0.36 (0.05-0.65)	0.461
**ALP(IU/L)**	71.5 (69-74)	75 (70-89)	75 (70-99)	**0.002** ^*^
**CRP (mg/L)**	9 (4-14)	30 (3-130)	57.5 (3-140)	**0.026** ^*^
**Albumin in urine (g/dL)**	2 (1-3)	2 (1-4)	3.5 (1-4)	0.057
**Glucose in urine (mmol/L)**	0.5 (0-1)	0 (0-1)	0 (0-1)	0.085
**Urea (mg/dL)**	18 (12-24)	24 (11-44)	27 (18-35)	0.112
**Serum creatinine(mg/dL)**	1.15 (0.9-1.4)	0.68 (0.5-1.5)	0.72 (0.6-1.1)	**0.002** ^*^
**Serum uric acid (mg/dL)**	3.75 (3.1-4.4)	3.7 (3-5.1)	3.95 (3.5-7.0)	0.399
**FBS (mg/dL)**	84.5 (75-94)	73.5 (60-99)	82.5 (65-100)	0.079
**2 h PP(mg/dL)**	113.5 (112-115)	121.5 (95-140)	124.5 (110-145)	0.399
**PT (s)**	12.5 (12-13)	12 (11-13)	13 (12-14)	**<0.0001** ^*^
**PC (mg/L)**	95 (80-110)	90 (80-150)	115 (70-140)	0.424
**PTT (s)**	27 (24-30)	34.0 (24-45)	35.0 (25-44)	**0.017** ^*^
**INR**	0.9 (0.9-0.9)	1 (0.8-1.0)	0.95 (0.8-1.0)	**0.021** ^*^
	**N (%) **			**P-value** ^##^
**Mild**	4 (50.0)	20 (41.7)		0.659
**Severe**	4 (50.0)	28 (58.3)	8 (33.3)	

#: Statistical analyzes were performed by Kruskal-Wallis and Chi-squared (χ2) test.*: Statistically significant.

**Table 5:** Pregnancy and delivery characteristics in relation to genotypes in preeclampsia cases

**Variables**	**TT (N=8)**	**TA (N=48)**	**AA (N=24)**	**P-value** ^#^
	**Median (range)**			
**Gestational age(weeks)**	39.5 (39-40)	36.75 (34-40)	36.00 (34-39)	**<0.0001** ^*^
**Fetal birth weight (Kg)**	3.1 (2.8-3.4)	3.05 (1.8-3.7)	2.8 (1.7-3.2)	**0.013** ^*^
	**N (%)**			**P-value** ^##^
**Abnormal doppler**	0 (0.0)	12 (25.0)	16 (66.7)	----
**Reduced amniotic fluid**	0 (0.0)	20 (41.7)	20 (83.3)	----
**IUGR**	4 (50.0)	12 (25.0)	16 (66.7)	**0.003** ^*^
**Vaginal** **CS**	4 (50.0) 4 (50.0)	32 (66.7) 16 (33.3)	20 (83.3) 4 (16.7)	0.149

IUGR: intra uterine growth retardation; CS: cesarean section; #: statistical analyzes were performed by Kruskal-Wallis and Chi-squared (χ2) test.*: statistically significant.

**Table 6:** Comparison between genotypes regarding the relativemiR-155 expression levels in preeclampsia cases

**Variables**	**TT (N=8)**	**TA (N=48)**	**AA (N=24)**	**P-value** ^#^
	**Median (range)**			
**Relative expression levels of miR-155 in cases**	3.70 (2.37-5.03)	7.47 (0.09-12.15)	3.41 (2.00-8.62)	<0.0001* TT-TA 0.007* TT-AA 1.000* TA-AA <0.0001*

## Discussion

During normal pregnancy, the placenta expresses different miRNAs according to gestational age (15). A major source of placental miRNAs is the villous trophoblasts (16,17). Hypoxia plays an important role in their activity(18). For example, mir-146a and mir-223 are known to be dysregulated in PE, and interact with many immune cells such as macrophages and dendritic cells (19).Mir-155 is another miRna that has been investigated widely in several immunological disorders (20). It is processed in humans from exon 3 of the non-protein coding B-cell integration cluster (*BIC*) RNA (21). Its expression is induced in activated B-cells, T-cells, and macrophages and several studies have found it overexpressed in several types of B-cell lymphoma (22).

Mir-155 has emerged as an inflammatory-related miRNA, as it can be significantly up-regulated by tumor necrosis factor-aand lipopolysaccharide (23). There is a strong association between mir-155 and pathogenesis of PE due to inflammation (24). O’Connell et al. found that mir-155 has been induced by toll-like receptors in macrophages, and acts as a target of many inflammatory mediators (25). Also, serum levels of mir-155 and interleukin-17A were found to increase in PE cases in comparison with controls (26).

In the current study, we found that there was a significant difference between the cases and controls regarding the expression level of mir-155 with up-regulation in PE patients (5.86 ± 3.11) (P<0.0001). Zhang et al. for the first time found that mir-155 contributed to PE by downregulating cysteine -rich 61(*CYR61*) geneby targeting a region within its 3′UTR which leads to decreased levels of *CYR61* in PE placentas. CYR61 is an important angiogenic regulating factor during pregnancy, and is essential for vascular integrity by inducing the expression of vascular endothelial growth factor (VEGF) (24). The decrease in the expression of VEGF causes reduced angiogenesis,and therefore placental undeperfusion, leading to PE initiation (27).

In addition, Cheng et al. found that mir-155 regulates angiotensin II type 1 receptor expression in umbilical vein endothelial cells from women with PE. Angiotensin II by inducing low-grade inflammation on endothelial, vascular, and immune cells could explain its role in the pathogenesis of PE (28).

Moreover, it was validated that the over-expression of mir-155 decreased endothelial nitric oxide synthase expression and NO production(29). Our results are consistent with the previous study of Li et al. who found high expression levels of mir-155 in PE patients in comparison with controls by regulating nitric oxide synthase(30).

Genetic variants in the functional elements of miRNAmay affect its expression, maturation or mRNA recognition, and alter disease susceptibility (31). Rs767649 T > A polymorphism of mir-155 has recently been studied in many diseases (11- 13) and for the first time we searched its role in PE. The results showed that the minor allele of rs767649 was significantly associated with increased risk of PE,and A allele was significantly associated with PE risk, compared with T allele. Also, there were significant differences between different genotypes regarding mir-155 expression level in PE (P<0.0001) with a high level in TAgenotype.

Diagnostic performance analysis of mir-155 showed its diagnostic value to differentiate PE patients from healthy control subjects as follows: AUC = 0.950, P < 0.0001, cutoff point 1.57, 95.0% sensitivity, and 100.0% specificity (Figure 1) which revealed that the relative expression level of mir-155 could be used as a potential biomarker for PE diagnosis and prognosis, and also as a promising management tool.

In conclusion, mir-155 may play a critical role in PE pathogenesis. The obtained data suggest that the minor allele of rs767649 might be a predisposing factor for PE.

## Conflict of interest

Authors declare no conflict of interest.
